# Feasibility of a brief group-based immersive 360° mindfulness program for stress-related outcomes in health sciences students: a 4-week pre–post pilot study

**DOI:** 10.3389/fmed.2026.1740585

**Published:** 2026-02-25

**Authors:** Gabriel Roma, Oriol Yuguero

**Affiliations:** 1 Facultad de Medicina, Universidad de Lleida (UdL-IRBLLEIDA), Lleida, Spain; 2 eHealth Center, Universitat Oberta de Catalunya (UOC), Barcelona, Spain

**Keywords:** feasibility study, health sciences students, immersive 360° projection, mindfulness, perceived stress

## Abstract

**Background:**

Medical and nursing students experience high stress that can impair well-being and training. Immersive technologies may offer brief, scalable relaxation. MK360 combines short guided mindfulness with immersive 360° projection in a shared multisensory room environment without wearables.

**Objective:**

To assess feasibility and explore short-term changes in perceived stress and immediate autonomic arousal following a brief, predominantly group-based immersive 360° mindfulness program in medical and nursing students.

**Methods:**

Single-site pre–post pilot at the University of Lleida. Students attended four ~20-min MK360 sessions over 4 weeks. Heart rate (HR) and blood pressure (SBP/DBP) were recorded immediately before and after each session. The Perceived Stress Scale (PSS-14), the Goldberg Anxiety and Depression Scale-anxiety subscale (GADS/EADG), and the Maslach Burnout Inventory-Student Survey (MBI-SS) were administered at baseline and endline. Pre–post changes were tested using Wilcoxon signed-rank tests (*α* = 0.05); a one-tailed test was pre-specified for PSS (direction: decrease).

**Results:**

Twenty-six students initiated the study (mean age 21.0 years; 88.9% female; 83.3% medical students), and 18 completed all assessments (completion 69.2%). Perceived stress decreased from baseline to endline (PSS-14 median [IQR] 29.5 [26.0–33.5] to 26.5 [19.5–33.8]; *Δ* − 3), but did not reach statistical significance in the pre-specified one-tailed Wilcoxon test (p ≈ 0.07; two-tailed sensitivity p ≈ 0.15; *r* ≈ −0.35). Burnout subscales showed non-significant directional changes (exhaustion and cynicism decreased; academic efficacy increased). Anxiety scores showed no material change. Immediate pre- to post-session reductions were observed for HR (mean *Δ* − 1.8 to −5.5 bpm) and SBP (Δ − 2.7 to −8.4 mmHg) across sessions; DBP decreased in sessions 2 and 4 but not in sessions 1 and 3. Among completers, 50.0% reported feeling somewhat better emotionally and 38.9% no change. No serious adverse events were reported.

**Conclusion:**

MK360 was feasible and well tolerated, with signals consistent with reduced perceived stress over 4 weeks and transient reductions in cardiovascular arousal within sessions. Given the uncontrolled design and small sample, causal inference is not warranted; controlled studies should confirm effectiveness, examine dose–response, and compare delivery formats.

## Introduction

Burnout and stress are highly prevalent among health sciences students and are linked to poorer mental health, reduced empathy, and attrition from clinical careers (1). Recent meta-analytic evidence indicates a substantial burden of burnout in both medical students and nursing students, supporting the need for prevention strategies during training (2, 3). This population is subjected to high academic demands, early exposure to emotionally demanding clinical situations, and a competitive learning environment. As a result, many students develop significant symptoms of chronic stress, anxiety, and emotional disconnection, which negatively impact their well-being, academic performance, and future careers (4, 5).

Beyond documenting prevalence, recent evidence has examined whether brief, scalable, technology-enabled interventions can buffer stress during training (6). Mindfulness-based interventions have shown potential benefits in medical education, although results are heterogeneous and effects on burnout and anxiety are not consistently observed across studies (7). Immersive virtual reality (IVR) offers controlled, multimodal environments that may elicit rapid relaxation and attentional disengagement from stressors through plausible biological and psychological mechanisms. From a mechanistic standpoint, IVR relaxation may trigger the relaxation response and autonomic down-regulation (parasympathetic activation), observable as reductions in heart rate and blood pressure and increases in heart-rate variability (HRV). Experimental and quasi-experimental studies show that immersive content can acutely lower cardiovascular arousal and modulate autonomic reactivity, supporting biological plausibility for short-term stress relief (8). Psychologically, IVR can provide guided attention and cognitive distraction, immersive nature exposure, and mindfulness scaffolding that together reduce perceived stress. These mechanisms have been leveraged in trials and feasibility studies across students and healthcare workers (9, 10).

Syntheses of IVR for stress and well-being in young adults and students report mixed but generally favorable short-term effects on perceived stress and relaxation, while noting heterogeneity of protocols and small samples (11–13). A 2024 systematic review in JMIR Mental Health found modest benefits on stress and well-being with substantial variability and risk of bias (14). Device-assisted mindfulness studies suggest comparable or superior engagement and physiological changes when mindfulness is delivered in VR versus flat-screen formats (15). Recent university-based and general population studies also indicate improvements in mood and stress with brief, nature-based or mindfulness IVR sessions, though effects on anxiety and burnout are less consistent and often require higher “dose” and longer follow-up (16). Studies have analysed physiological stress responses during VR-based clinical simulations, supporting the relevance of immersive contexts for emotional preparation. These studies highlight the potential of VR to emotionally prepare students for demanding clinical contexts.

Against this backdrop, we conducted a single-site pilot to explore whether a brief, four-session IVR program is feasible and associated with short-term reductions in perceived stress and immediate post-session decreases in physiological arousal among medical and nursing students during clinical rotations (17, 18). This work aims to address gaps identified by reviews—namely, the need for pragmatic protocols reporting both psychometric and physiological outcomes, alongside feasibility and tolerability data. The objective of the research team was to assess feasibility and short-term changes in (a) perceived stress and (b) immediate physiological arousal (heart rate, systolic/diastolic blood pressure) associated with a brief, four-session immersive virtual reality relaxation program in health sciences students at a single institution, using a pre–post uncontrolled pilot design.

## Materials and methods

### Design and setting

We conducted a single-site, uncontrolled pre–post pilot study at the Faculty of Medicine, University of Lleida (Lleida, Spain). The intervention was delivered using the MK360 immersive projection system in a dedicated room at the university facilities.

### Ethical approval and consent

The study protocol was reviewed and approved by the Clinical Research Ethics Committee of IRBLleida (Institut de Recerca Biomèdica de Lleida), Lleida, Spain (approval code/reference: CEIC 2878; date of approval: June 24, 2023). All participants provided written informed consent prior to participation. The study was conducted in accordance with applicable ethical standards and data protection regulations.

### Participants, eligibility, and recruitment

#### Eligibility criteria

Participants were medical and nursing students aged ≥18 years enrolled at the University of Lleida and able to provide informed consent. Exclusion criteria were: history of epilepsy or photosensitive seizures; severe motion sickness; current vestibular disorder; pregnancy-related restrictions according to institutional guidance; and any other condition deemed incompatible with exposure to immersive audiovisual stimulation.

#### Recruitment procedures and sample flow

Recruitment used a convenience sampling strategy. Invitations were distributed by email and reinforced through brief informational sessions. Specifically, emails were sent to all medical and nursing students in the 1st and 3rd year for nurses and the 5th for medical students. Eligibility screening was based on email reply and checklist.

A total of 82 students were contacted by email, 35 responded, and 28 met eligibility criteria.26 provided consent and initiated the program, and 18 completed endline assessments. Reasons for non-participation or attrition were recorded when available (e.g., scheduling conflicts, withdrawal, incomplete follow-up).

#### Sample size rationale

No formal sample size calculation was performed given the pilot nature of the study. An *a priori* target of approximately 25 participants was set to estimate feasibility parameters (recruitment, adherence, retention) and the variability of key outcomes to inform future controlled studies.

### Intervention (MK360 immersive 360° mindfulness program)

Participants were offered four sessions over 4 weeks (target dose: one session per week), each lasting approximately 20 min. Sessions were self-scheduled to accommodate academic duties and clinical rotations.

Each session followed a standardized structure:

1. Guided mindfulness (≈5 min): audio-guided attention to breathing and present-moment awareness.
2. Immersive 360° projection (≈15 min): immersive audiovisual content accompanied by non-verbal ambient music.

To minimize habituation and explore acceptability of different restorative environments, the 360° visual content varied across weeks: (i) natural landscapes, (ii) beach/ocean scenes, (iii) abstract/psychedelic imagery, and (iv) an urban cityscape. Sessions could be completed individually or in small groups (maximum 4 students). In practice, most sessions were completed in small groups (approximately 90%).

Standardized instructions (room setup, safety precautions, and mindfulness guidance) were provided via a QR code accessible at the intervention site. Participants were advised to stop the session if they experienced discomfort.

### Outcomes and measurements

#### Primary outcome

The primary outcome was change in perceived stress measured by the Perceived Stress Scale (PSS-14) from baseline (pre-intervention) to endline (after the 4-week program). As the PSS assesses stress during the previous month, endline responses were interpreted as reflecting perceived stress across the intervention window rather than acute session effects.

Language/validation: The PSS-14 was administered using a validated Spanish-language version (19).

#### Secondary outcomes

1. Immediate autonomic arousal markers: pre- to post-session change in heart rate (HR) and blood pressure (SBP/DBP) recorded immediately before and after each session.
2. Anxiety symptoms: baseline to endline change using the Goldberg Anxiety and Depression Scale – anxiety subscale (GADS/EADG) (9 dichotomous items; total 0–9; higher scores indicate more symptoms; ≥4 suggesting probable anxiety).
3. Burnout symptoms: baseline to endline change using the Maslach Burnout Inventory–Student Survey (MBI-SS) (15 items; subscales Exhaustion, Cynicism, Academic Efficacy).
4. Participant-reported emotional change: a single-item self-report at endline describing perceived emotional change (e.g., “much better/somewhat better/no change/somewhat worse/much worse”)

Language/validation: Validated Spanish-language versions were used for GADS/EADG and MBI-SS (20, 21).

#### Physiological measurements (HR and BP)

HR, SBP, and DBP were obtained using an automated upper-arm blood pressure monitor (OMRON). Measurements were taken in a seated position after a brief seated rest. Cuff size was adapted to arm circumference. Each timepoint consisted of a single reading. Measurements were collected immediately before and immediately after each session.

Potential acute confounders (e.g., caffeine intake, sleep, time-of-day, medication, physical activity) were not systematically recorded and are addressed as limitations.

#### Safety and tolerability (cybersickness)

Adverse effects were monitored after each session using a brief cybersickness checklist. This checklist was developed *ad hoc* for this pilot. It comprised 5 items assessing common symptoms (nausea, dizziness, headache, visual discomfort and disorientation) rated on [none/mild/moderate/severe]. Participants were instructed to discontinue the session if symptoms were moderate-to-severe, and any events were recorded. No adverse events occurred.

### Statistical analysis

Continuous variables are summarized as median (interquartile range, IQR) and categorical variables as counts and percentages. Pre–post changes were evaluated using the Wilcoxon signed-rank test (two-sided *α* = 0.05), after inspection of paired differences to support a non-parametric approach in this pilot sample. For each comparison, effect size was reported as *r* = *Z*/√*n* (where *n* is the number of non-zero paired differences). Change estimates are provided as the Hodges–Lehmann median paired difference with 95% confidence intervals estimated via bootstrap resampling. Analyses were performed using SPSS (v28.0; IBM Corp.). Missing data were handled using complete paired observations for each outcome (available-case analysis; no imputation).

## Results

### Participants and baseline characteristics

A total of 26 students initiated the study and 18 completed the full 4-session program, yielding a completion rate of 69.2%. Table 1 summarizes participants’ characteristics stratified by study status (initiators vs. completers); baseline characteristics were broadly comparable between groups. Among completers, the mean age was 21.0 years (SD 2.8) and 88.9% were women. Most were medical students (83.3%) and the remainder nursing students (16.7%); participants were enrolled in first year (66.7%) or fifth year (33.3%). Most reported no history of anxiety/depression (72.2%), whereas 27.8% reported a history of anxiety; 5.6% were under treatment.

**Table 1:** Baseline characteristics of participants (initiators vs. completers).

Sociodemographic variables	Participants	
	Initiators	Completers
	*N* = 26	*N* = 18
Age mean (SD)	20.6 (2.8) Min 18–Max 27	21.0 (2.8) Min 18–Max 27
Gender (female)	76.9% (20)	88.9% (16)
Student category		
Nurse students	26.9% (7)	16.7% (3)
Medical students	73.1% (19)	83.3% (15)
Academic year		
1st	69.2% (18)	66.7% (12)
3rd	7.7% (3)	0.0% (0)
5th	23.1% (6)	33.3% (6)
Anxiety/depression background		
No	76.9% (20)	72.2% (13)
Anxiety	23.1% (6)	27.8% (5)
Depression	0.0% (0)	0.0% (0)
Both	0.0% (0)	0.0% (0)
Under treatment (Yes)	3.9% (1)	5.6% (1)
Emotional change at the end		
No changes	n.a.	38.9% (7)
Somewhat better	n.a.	50.0% (9)
Much better	n.a.	5.6% (1)
Somewhat worse	n.a.	5.6% (1)
Much worse	n.a.	0.0% (0)

Values are expressed as mean (SD) for continuous variables and as percentage (frequency) for categorical variables. n.a., not applicable; n.s., not significant.

#### Burnout, perceived stress, and anxiety

Pre–post changes among completers (*n* = 18) are reported in Table 2 using medians (IQR). Emotional exhaustion decreased from baseline to endline (median [IQR] 18.5 [15.2–22.0] to 16.0 [14.0–19.0]; *Δ* − 2), reaching borderline significance in two-tailed Wilcoxon testing (*p* = 0.054). Cynicism showed a reduction (10.0 [7.2–13.8] to 6.0 [5.2–10.0]; *p* = 0.108), while academic efficacy increased (25.5 [20.2–26.0] to 27.0 [22.2–30.0]; *p* = 0.167).

**Table 2:** Burnout, stress and anxiety relation with IRV.

MBI-SS (median IQR)	Baseline	Post	Δ (post–pre)	*p*
Exhaustion	18.5 (15.2–22.0)	16.0 (14.0–19.0)	−2	0.05
Cynism	10.0 (7.2–13.8)	6.0 (5.2–10.0)	−1	0.108
Academic efficacy	25.5 (20.2–26.0)	27.0 (22.2–30.0)	+3	0.16
PSS median (IQR)	29.5 (26.0–33.5)	26.5 (19.5–33.8)	−3	0.07
GADS median (IQR)	5.0 (3.2–6.8)	5.5 (1.5–7.0)	0	0.972

PSS primary comparison tested one-tailed (pre-specified direction: decrease). All other comparisons two-tailed.

Perceived stress (PSS-14) decreased from baseline to endline (29.5 [26.0–33.5] to 26.5 [19.5–33.8]; *Δ* − 3). In line with the pre-specified directional hypothesis (post < baseline), the one-tailed Wilcoxon signed-rank test yielded p ≈ 0.07 (two-tailed sensitivity p ≈ 0.15), with a small-to-moderate effect size (r ≈ −0.35). Goldberg anxiety scores did not change materially over time (Table 2).

#### Vital signs during sessions

Table 3 summarizes pre- and post-session heart rate and blood pressure across the four sessions. Post-session values were generally lower than pre-session values, with statistically significant reductions in heart rate and systolic blood pressure in all sessions, and in diastolic blood pressure in sessions 2 and 4 (Table 3). Table 4 provides descriptive summaries of within-session changes (post–pre), showing a consistent decrease in heart rate and systolic blood pressure across sessions.

**Table 3:** Vital signs recorded in each session.

Session	HR pre	HR post	SBP pre	SBP post	DBP pre	DBP post
1	81.3 (13.5)	77.8 (11.7)^*^	113.3 (13.7)	107.8 (10.6)^*^	74.2 (7.1)	74.6 (4.5)
2	75.7 (11.5)	73.8 (10.3)^*^	112.6 (9.9)	104.2 (9.7)^*^	72.4 (7.3)	66.6 (7.3)^*^
3	78.1 (12.9)	73.8 (11.0)^*^	110.4 (11.9)	104.2 (11.8)^*^	69.2 (6.5)	67.7 (8.8) n.s
4	79.2 (9.3)	73.7 (9.6)^*^	108.8 (8.0)	106.1 (8.4)^*^	72.3 (7.8)	66.8 (7.4)^*^

Values are expressed as mean (SD). HR, heart rate; SBP, systolic blood pressure; DBP, diastolic blood pressure. *p < 0.05 when comparing pre- and post-intervention values using paired *t*-test; n.s., not significant.

**Table 4:** Descriptive analysis of post-pre differences in vital signs measured before and after each session.

Session	Δ HR	Δ SBP	Δ DBP
1	−5.5 (5.4)	−5.4 (9.0)	0.3 (4.1)
2	−1.8 (4.1)	−8.4 (10.4)	−5.9 (6.3)
3	−4.3 (6.8)	−6.2 (8.8)	−1.6 (6.1)
4	−5.5 (8.4)	−2.7 (5.8)	−5.6 (5.2)

Values are expressed as mean (SD). HRΔ, post-pre difference in heart rate; SBPΔ, post-pre difference in systolic blood pressure; DBPΔ, post-pre difference in diastolic blood pressure.

Self-reported emotional change. At endline, 50.0% of completers reported feeling somewhat better emotionally and 38.9% reported no change; 5.6% reported feeling much better and 5.6% somewhat worse (Table 1).

## Discussion

In this single-site, uncontrolled pre–post pilot, a brief four-session immersive 360° mindfulness program (guided mindfulness plus 360° projection) was feasible to implement within a health sciences curriculum. Allowing participants to self-schedule sessions accommodated heterogeneous academic and clinical timetables, and delivery in small groups may have supported adherence and completion.

Over the 4-week period, we observed a signal of reduced perceived stress (PSS-14), whereas anxiety and burnout subscales did not show statistically significant changes. In parallel, heart rate and blood pressure showed small immediate pre–post reductions within sessions, consistent with an acute relaxation response. This pattern aligns with a growing body of research indicating that VR-based relaxation, mindfulness, and virtual nature exposures can reduce stress and distress—often with small-to-moderate short-term effects and substantial heterogeneity across populations, content, and outcome measures (22, 23).

Mechanistically, immersive environments may facilitate attentional absorption and perceived “presence,” supporting downregulation of arousal via parasympathetic activation. Experimental and comparative work has linked relaxing VR exposure to changes in autonomic markers and stress-related outcomes, sometimes with profiles comparable to established physiological relaxation approaches (24, 25). A recent meta-analysis focusing on virtual natural environments reported significant reductions in stress in healthy adults, although heterogeneity was high—highlighting that effects depend on both the stimulus and the recipient context (23).

### Why perceived stress may change while burnout and anxiety do not

The partial dissociation observed here—signals on global perceived stress but not on burnout or anxiety—may be expected given the brief dose, the pilot sample size, and the constructs involved. The PSS captures a global appraisal of stress over the preceding month, which overlaps temporally with the intervention window and may be relatively responsive to low-burden interventions. In contrast, burnout reflects more cumulative processes (exhaustion and disengagement) that may require greater intensity, longer duration, or additional components targeting workload, recovery, and meaning at work/study. This is particularly relevant in health sciences education, where stressors are episodic and context-dependent, peaking during clinical exposures and performance evaluation. Evidence from university mindfulness research more broadly supports modest improvements in stress and wellbeing, with variable effects on anxiety and other outcomes depending on design, dose and follow-up (17, 26).

### Physiological findings and psychobiological framing

The consistent within-session decreases in heart rate and systolic blood pressure observed across sessions support an interpretation of acute autonomic calming during/after exposure. However, vital signs are coarse markers and can be influenced by posture, measurement timing, and situational factors. These findings align with experimental work indicating that immersive VR exposures can modulate physiological responses and may influence autonomic arousal (27), and with emerging evidence that digital and VR-based stress-reduction interventions can be feasible and potentially beneficial in high-demand health-related contexts, albeit with heterogeneity and pilot-level uncertainty (28, 29) Prior work suggests that stress-related physiological systems may change asynchronously (e.g., autonomic vs. endocrine), and short interventions may produce detectable changes in some proximal markers without parallel shifts in broader symptom scales (30). Recent studies using more sensitive measures such as heart rate variability (HRV) indicate that virtual nature can enhance parasympathetic markers (e.g., SDNN), reinforcing the plausibility of autonomic pathways for VR-based relaxation (25, 31).

Interpretation benefits from a broader psychobiological framework of stress in which short-term autonomic responses (e.g., heart rate and blood pressure) and endocrine pathways (e.g., HPA-axis activity) may not change in tandem and can be partially dissociated, particularly in brief interventions (32). Accordingly, acute reductions in cardiovascular arousal may coexist with limited or delayed changes in broader symptom measures. Differences in baseline stress reactivity, temperament, and coping styles may also help explain heterogeneity in subjective responses, as suggested by work linking perceived stress with cortisol dynamics and individual traits in student populations (33).

### Limitations

This pilot has important limitations. First, the uncontrolled pre–post design restricts causal inference and remains vulnerable to regression to the mean, history effects, and expectancy. Second, potential acute confounders (sleep, caffeine, medication, physical activity, and time-of-day) were not systematically measured. Third, the PSS reflects the prior month; therefore, changes should be interpreted as a global perceived-stress signal across the intervention window rather than a direct acute effect of any single session. Fourth, physiological assessment was limited to vital signs without HRV or endocrine indicators, and we did not collect qualitative feedback that could have strengthened acceptability and mechanism interpretation.

A further consideration is that most sessions were delivered as a shared experience. Group delivery may influence outcomes through social context (e.g., perceived safety, peer effects, distraction), introducing heterogeneity relative to individual VR paradigms. Future studies should prospectively standardize delivery format (individual vs. group), measure expectancy and engagement, and use analytic approaches that characterize heterogeneity (e.g., baseline stratification and mixed-effects models).

### Implications and future directions

Future research should prioritize controlled designs (preferably randomized), with pre-specified analytic plans and outcomes spanning: (i) state and trait psychometrics, (ii) autonomic measures with higher resolution (e.g., HRV), (iii) endocrine indicators (e.g., diurnal salivary cortisol), and (iv) qualitative evaluation of the participant experience. These additions would clarify mechanisms, identify subgroups most likely to benefit, and support decisions about optimal content, dosing, and delivery format. Trials in student populations may also benefit from aligning timing with known high-stress periods (e.g., examinations or clinical rotation transitions), where signal-to-noise may be greater.

## Conclusion

In a small, single-site pre–post pilot, a brief four-session immersive 360° mindfulness program was feasible and showed signals consistent with reduced perceived stress over 4 weeks and transient decreases in autonomic arousal within sessions. Controlled trials incorporating state measures, qualitative evaluation, and autonomic/endocrine indicators are needed to confirm effectiveness, clarify mechanisms, and define optimal content, dosing, and delivery format.

## Data Availability

The original contributions presented in the study are included in the article/supplementary material, further inquiries can be directed to the corresponding author.
